# Massive parallel sequencing in a family with rectal cancer

**DOI:** 10.1186/s13053-021-00181-2

**Published:** 2021-04-07

**Authors:** Karin Wallander, Jessada Thutkawkorapin, Ellika Sahlin, Annika Lindblom, Kristina Lagerstedt-Robinson

**Affiliations:** 1 Department of Molecular Medicine and Surgery, Karolinska Institutet, Solna, Stockholm Sweden; 2 Department of Clinical Genetics, Karolinska University Hospital, Solna, Stockholm Sweden

**Keywords:** Rectal cancer genetics, Massive parallel sequencing, WGS, WES, CENPB

## Abstract

**Background:**

We have previously reported a family with a suspected autosomal dominant rectal and gastric cancer syndrome without any obvious causative genetic variant. Here, we focused the study on a potentially isolated rectal cancer syndrome in this family.

**Methods:**

We included seven family members (six obligate carriers). Whole-exome sequencing and whole-genome sequencing data were analyzed and filtered for shared coding and splicing sequence and structural variants among the affected individuals.

**Results:**

When considering family members with rectal cancer or advanced adenomas as affected, we found six new potentially cancer-associated variants in the genes *CENPB*, *ZBTB20*, *CLINK*, *LRRC26*, *TRPM1*, and *NPEPL1.* All variants were missense variants and none of the genes have previously been linked to inherited rectal cancer. No structural variant was found.

**Conclusion:**

By massive parallel sequencing in a family suspected of carrying a highly penetrant rectal cancer predisposing genetic variant, we found six genetic missense variants with a potential connection to the rectal cancer in this family. One of them could be a high-risk genetic variant, or one or more of them could be low risk variants. The p.(Glu438Lys) variant in the *CENPB* gene was found to be of particular interest. The CENPB protein binds DNA and helps form centromeres during mitosis. It is involved in the WNT signaling pathway, which is critical for colorectal cancer development and its role in inherited rectal cancer needs to be further examined.

**Supplementary Information:**

The online version contains supplementary material available at 10.1186/s13053-021-00181-2.

## Background

Colorectal cancer has the third highest cancer incidence worldwide and it is the second most deadly form of cancer [1]. It is estimated that 35% of all colorectal cancer cases are due to an inherited predisposition [2]. In most cases, the genetics behind the heritability is unknown. With improving techniques for massive parallel sequencing, knowledge on the genetics behind colorectal cancer is believed to increase.

We have previously published results from linkage and exome analyses in a family with multiple cases of rectal and gastric cancer [3, 4]. Twelve novel potentially damaging sequence variants were reported, possibly contributing to the increased risk of cancer in this family. The family pedigree clearly indicates an autosomal dominant, highly penetrant disease, and since the previous study one additional individual has been diagnosed with rectal cancer. This prompted us to re-analyze the family and to focus on rectal cancer as a separate entity. Whole-genome sequencing (WGS) or whole-exome sequencing (WES) were performed on six samples, including a recently affected individual. In addition, we performed array-CGH, as well as a structural variant analysis using WGS data [5], in order to search for deletions, duplications, inversions or other chromosomal rearrangements.

## Methods

### Family description

In this family (Family no 242), multiple individuals in four generations have had rectal or gastric cancer. The family was included in the study after one of the family members was referred to the Department of Clinical Genetics, Karolinska University Hospital, Solna, Stockholm, Sweden, for genetic counselling. Three siblings with rectal cancer and one sibling with large tubulovillous adenomas with high-grade dysplasia in the rectum were included (I1, I2, I3, I4), as can be seen in Table 1. Microsatellite instability testing had been performed on tumor material from individual I1 with normal results. All family members alive at the time of inclusion in the study and affected by verified rectal cancer or advanced polyps were included. The father of the siblings had gastric cancer and the mother of the siblings had colorectal cancer. There were also three other siblings. None of these five individuals were alive or had a verified cancer diagnosis at the time of inclusion into the study. Therefore, it was not possible to obtain DNA samples from them. Also included in the study, and considered affected, were a daughter (II1) and a son (II2) to one of the siblings (I2). Included in the sequencing, but not considered necessarily affected, was also another sibling (I5), with four rectal tubular adenomas. There was no other sibling than the ones described above, and all children of the siblings were un-affected at the time of the study.

**Table 1:** All family members included in the genetic analyses, their cancer diagnoses and age at diagnosis

Individual	Diagnosis	Age
I1	Rectal cancer	63
I2	Rectal cancer	50
I3	Rectal cancer	63
I4	3 rectal tubulovillous adenomas with high grade dysplasia (CIS), > 10 mm	62
II1	Rectal cancer	63
II2	Rectal cancer	42

*CIS* cancer in situ

### Samples and massive parallel sequencing

Blood samples were collected and DNA was isolated according to standard protocols. WES data from the previous study was available from three of the relatives (I2, II2 and I5) [3]. WGS was performed on samples from the four additional family members (I1, I3, I4 and II1). Extracted DNA was converted to sequencing libraries using a PCR-free paired-end protocol (Illumina TruSeq DNA PCR-free). Sequencing was performed on the Illumina NovaSeq 6000 platform aiming at minimum 30x median coverage. WGS was performed at Clinical Genomics, SciLifeLab, Stockholm, Sweden.

Local variant allele frequencies were calculated from WES data from 98 anonymous individuals with colorectal cancer as well as 56 anonymous individuals with breast cancer. All these individuals had undergone genetic counselling at the Department of Clinical Genetics, Karolinska University Hospital, and had received a diagnosis of familial colorectal or breast cancer, according to family history and ages at cancer diagnoses.

### Variant annotation

The output in variant call format was annotated using ANNOVAR as previously reported [3]. Only exonic or predicted splice variants occurring either in a heterozygous or a homozygous state in the six family members were analyzed. Also, only variants with a minor allele frequency in publicly available cohorts [6–9] lower than 1% or lower than or equal to the frequency in the local colorectal cancer cohort were included. Variants occurring in the local breast cancer cohort were excluded.

The manual filtering process was complemented by the software MIP (Mutation Identification Pipeline) and visualization in Scout, as a secondary analysis pipeline [10], analyzing the four samples from the patients with WGS-data. Regarding the most recently affected individual in the family (II1), filtered shared variants detected in the family were checked manually in IGV (Integrated Genomics Viewer).

### Copy number variation/structural variants

Array-CGH analysis was performed on DNA from two participants (I1 and I3). The array-CGH was performed according to the manufacturer’s instructions and as part of the clinical procedure at the Department of Clinical Genetics, Karolinska University Hospital (1 M-array. Platform OGT Clinical Exome 1 M.) [11]. First, genome wide analysis of copy number variants ≥20 kb was performed using the CytoSure Interpret Software, version 4.10.41 (Oxford Gene Technology) with data aligned to the human reference sequence GRCh37/hg19. Secondly, a targeted analysis of genes associated to hereditary gastrointestinal cancer syndromes (*APC, MUTYH, EPCAM, MSH2, MSH6, MLH1, PMS2, BMPR1A, SMAD4, STK11, PTEN, POLD1, GREM1, GALNT12, MSH3, NTHL1, TP53* and *CDH1*) was performed using the same software. A similar analysis [5] was performed using WGS data on two affected individuals (I4 and II1).

## Results

### Sequencing

The six family members with rectal cancer or advanced adenomas of the rectum were considered obligate carriers of a variant associated with a rectal cancer syndrome. A putative isolated gastric cancer syndrome could not be further analyzed due to too few samples from family members with gastric cancer. Five variants in the genes *CENPB*, *CLINK*, *LRRC26*, *TRPM1*, and *NPEPL1* were found in all analyzed patients, as shown in Table 2.

**Table 2:** List of all variants with potential association to a rectal cancer syndrome*.* Shared variants in the six affected family members

Gene name	Gemomic position (GRCh37/hg19)	Nucleotide change	Amino acid change	dbSNP	Population frequency	In-house CRC cohort frequency	CADD score	I1	I2	I3	I4	II1	II2
*ZBTB20*	3:114069761	NM_001164342.2:	p.(Asp388Glu)	rs144663365	0.004976^a^	0.0076	22	het	het	het	het	het	het
		c.1164C > G											
*CLNK*	4:10509611	NM_052964.3:	p.(Val319Ala)	rs200431324	0.00009 ^b^	0.0076	21	het	het	het	het	het	het
		c.956 T > G											
*LRRC26*	9:140063557	NM_001013653.2:	p.(His252Tyr)	rs200591146	0.001613^a^	0.0096	17	het	het	het	het	het	het
		c.754C > T											
*TRPM1*	15:31294996	NM_001252020.1:	p.(Glu1320Lys)	rs117855013	0.00074 ^c^	0.0152	24	het	het	het	het	het	het
		c.3958G > C											
*CENPB*	20:3765819	NM_001810.5:	p.(Glu438Lys)	rs144899160	0.001903^a^	0.0086	20	het	het	het	het	het	het
		c.1312G > A											
*NPEPL1*	20:57287548	NM_024663.3:	p.(Asn305Ser)	rs202184995	0.003323^a^	0.0076	23	het	hom	hom	het	het	het
		c.914A > G											

*het* heterozygous, *hom* homozygous

^a^Data from the gnomAD database (the highest frequency of carriers in any population is stated) [9]

^b^Data from the ALFA study, dbSNP database [12]

^c^Data from the Page study, dbSNP database [12]

All variants except the one in the *ZBTB20* gene were also identified in the sibling I5. The mean sequencing depth was 32-35x in the WES data and 29-45x in the WGS data. The family member included in the previous study (a paternal cousin to the siblings in this study, with a diagnosis of gastric cancer) did not carry any of these variants (Supplementary Fig. 1, Additional file 1). All variants were missense and none of them were unique to this cohort. All variants were interpreted as variants of uncertain significance (VUS) according to the American College of Medical Genetics and genomics guidelines [13] and none of them had a consensus in silico prediction as pathogenic [14–17].

No additional variants could be detected by applying the software Scout to the data.

### Copy number variation

There were no detectable copy number variants or other chromosomal rearrangements in the DNA from the participants.

## Discussion

We have analyzed massive parallel sequencing data in seven family members from a family with a high probability of carrying a genetic high-risk variant predisposing to rectal cancer. Initially, we hypothesized that the family had a highly penetrant genetic variant associated with both rectal and gastric cancer. However, since we did not find any verified highly penetrant genetic variants associated to a gastric and rectal cancer syndrome [3], we switched hypothesis and instead considered the possibility that there are not one, but two different syndromes in the family; one associated to gastric cancer and the other to rectal cancer. The differences between this new study and the previous were that we only focused on individuals with rectal cancer and we could add one additional, recently affected, family member. We also included more samples in the local breast cancer data set used as comparison, and we re-analyzed all data using updated bioinformatic software tools. As a result, all of the variants from the previous study were excluded (Supplementary Fig. 1, Additional file 1), since they either did not occur in the new obligate carrier (eight variants in the genes *DZIP1L, PCOLCE2, IGSF10, SUCNR1, OR13C8, TAS2R7, SF3A1* and *TRIOBP*), or were present in the local breast cancer cohort (three variants in the genes *GAL3ST1, SEC16A* and *NOTCH1*), or did not pass the new quality control (one variant in the gene *EPB41L4B*).

In this analysis, we found six potentially cancer-associated variants. All variants were missense, and all occur in population databases although with a low frequency. The *CENPB* gene is the most interesting in the context of inherited rectal cancer, since it is part of the WNT signaling pathway [18]. Most, if not all, colorectal cancers show hyperactivation of the WNT pathway and it is believed to be the initiating and driving event in colorectal carcinogenesis [19]. This is the first reported cancer syndrome co-segregating with a variant in the *CENPB* gene [20]. The *CENPB* gene encodes the only centromeric protein with a sequence-specific DNA-binding function. It binds the CENP-B box within the centromere DNA [21] and facilitates centromere formation in interphase nuclei and on mitotic chromosomes. Centromeres associated with higher levels of CENP-B are less likely to mis-segregate than the others [22]. But the precise function and importance of CENP-B is still controversial and whether it has a role in aneuploidy in neoplastic phenotypes is not known [23].

When it comes to the other five genes selected in this study, none of them have a known connection to inherited cancer. Pathogenic inherited variants in the *ZBTB20* gene have a known association to Primrose syndrome, an autosomal dominant syndrome including intellectual dysfunction and specific morphological features, but no known cancer [24]. ZBTB is a transcriptional repressor and upregulation of ZBTB20 expression has been shown in gastric cancer tissue, while knock down leads to inhibited cell proliferation, migration and invasion. There have been discussions on the association of polymorphisms in the *ZBTB20* gene and risk of gastric cancer but no consensus has been reached [25]. ZBTB20 has also been shown to be involved in tumorigenesis of glioblastoma, liver cancer and lung cancer [26–28]. Since one of the siblings (I5) did not carry this variant, it is considered less likely to be associated to an increased risk for rectal cancer in the family. Also, the variant is classified as benign or likely benign in ClinVar by three different sources, none of which have submitted a phenotype or evidence details [29]. *CLNK, NPEPL1* and *LRRC26* have no known connection to inherited cancer syndromes [30, 31]. CLNK is involved in immunoreceptor signaling [30] and there are no reports on its potential role in tumorigenesis. NPEPL1 is a probable aminopeptidase [32] and it has been found to form a fusion with STX16 in cancer tissue [33]. Three family members are homozygous for the *NPEPL1* variant. We therefore consider it of less interest, as we suspect a dominant inheritance in the family. LRRC26 is reported to be a negative regulator of NF-κB activity and it is downregulated in triple-negative breast cancer [34]. Finally, *TRPM1* encodes a cation channel and pathogenic variants in the gene are associated to congenital night blindness, but no inherited cancer syndrome is described [35]. TRPM1 is, though, described as a marker of melanoma aggressiveness and low expression is related to higher invasiveness [36].

Genetic counselling has been offered to all family members, and first degree relatives to individuals with colorectal cancer or advanced polyps are offered regular colonoscopies and gastroscopies, with individual intervals. No pre-symptomatic clinical genetic testing is yet possible in this family.

## Conclusion

To summarize, we performed massive parallel sequencing in a family suspected of carrying a highly penetrant rectal cancer predisposing genetic variant. We found six missense variants with potential connection to the rectal cancer in this family. One of them could be a high risk genetic variant, or one or more of them could be low risk variants, each contributing but not being the only cause for the increased risk for rectal cancer in the family. We believe that the variant in the *CENPB* gene is the most interesting and that it could be connected to a rectal cancer syndrome. Further studies are needed to evaluate the involvement of the *CENPB* and the other genes in inherited rectal cancer.

## Supplementary Information

**Additional file 1: Supplementary Figure 1.** Comparison of the present and the previous study presented in a flowchart. At the top are all family members included in the studies. No variant remained from the previous study in the present study and none of the variants presented in the present study were selected in the previous study. Family member I5 was not considered an obligate carrier in the present study*. (PNG 180 kb)*

## Data Availability

The datasets analyzed in the current study are available from the corresponding author upon request.

## Abbreviations

WES: Whole-exome sequencing
WGS: Whole-genome sequencing
