# Supplementary figures and images for: Massive parallel sequencing in a family with rectal cancer
Source: Hered Cancer Clin Pract. 2021 Apr 7;19:23. doi: 10.1186/s13053-021-00181-2 (PMC8028209; doi:10.1186/s13053-021-00181-2)

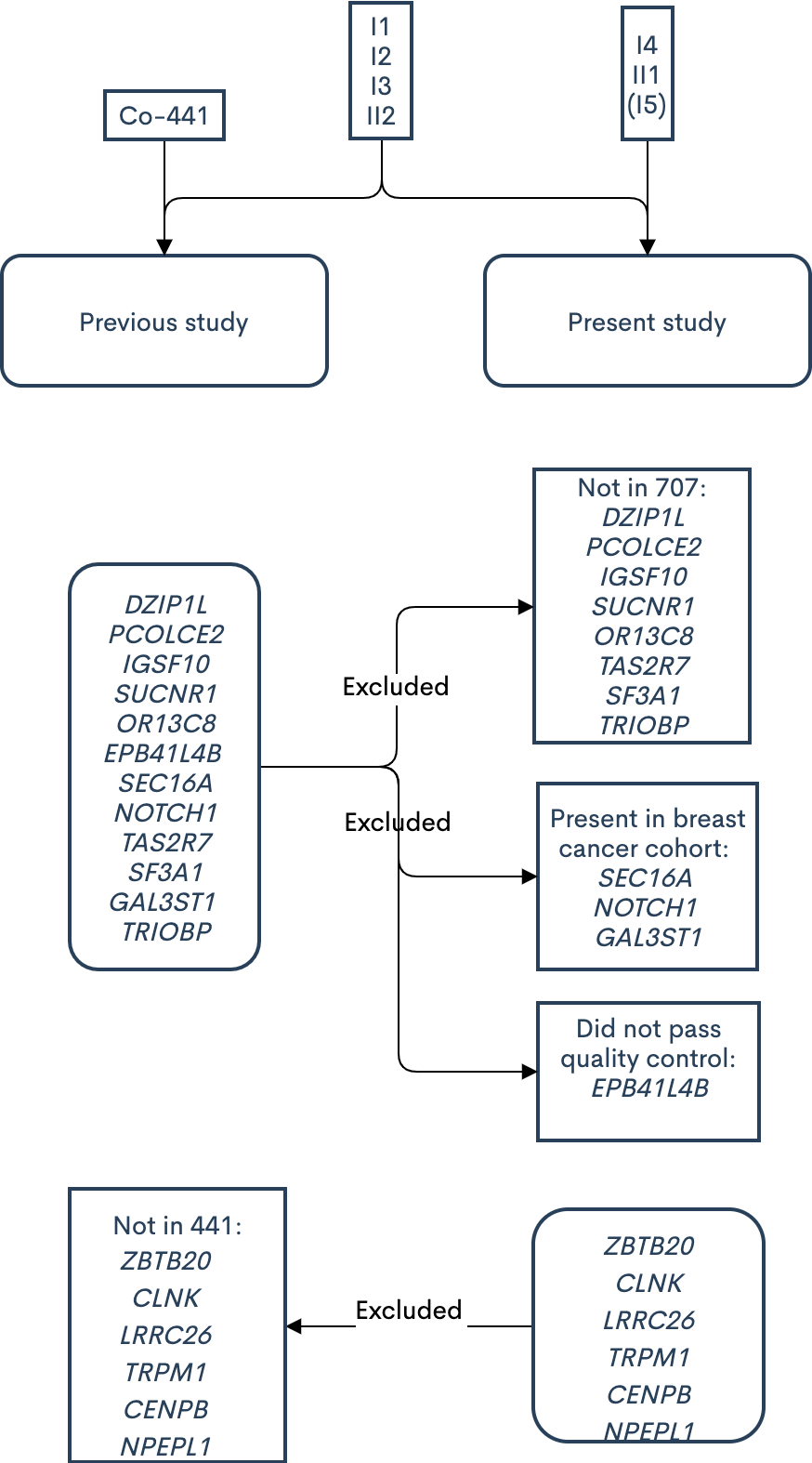

Supplement: Supplementary file 1 — Additional file 1: Supplementary Figure 1. Comparison of the present and the previous study presented in a flowchart. At the top are all family members included in the studies. No variant remained from the previous study in the present study and none of the variants presented in the present study were selected in the previous study. Family member I5 was not considered an obligate carrier in the present study. (PNG 180 kb) [file 13053_2021_181_MOESM1_ESM.png]
